# Infection with the Persistent Murine Norovirus Strain MNV-S99 Suppresses IFN-Beta Release and Activation of Stat1 *In Vitro*

**DOI:** 10.1371/journal.pone.0156898

**Published:** 2016-06-13

**Authors:** Sandra Niendorf, Uwe Klemm, Andreas Mas Marques, C.-Thomas Bock, Marina Höhne

**Affiliations:** 1 Department of Viral Gastroenteritis and Hepatitis Pathogens and Enteroviruses, Robert Koch- Institute, Berlin, Germany; 2 Max Planck Institute for Infection Biology, Berlin, Germany; Washington University School of Medicine, UNITED STATES

## Abstract

Norovirus infection is the main cause of epidemic non-bacterial gastroenteritis in humans. Although human norovirus (HuNoV) infection is self-limiting, it can persist for extended periods of time in immune deficient patients. Due to the lack of robust cell culture and small animal systems, little is known about HuNoV pathogenicity. However, murine norovirus (MNV) can be propagated in cell culture and is used as a model to study norovirus infection. Several MNV are known to persist in mice. In this study, we show that the MNV strain MNV-S99 persists in wild type inbred (C57BL/6J) mice over a period of at least 5 weeks post infection. Viral RNA was detectable in the jejunum, ileum, cecum, and colon, with the highest titers in the colon and cecum. To characterize the effect of MNV-S99 on the innate immune response, Stat1 phosphorylation and IFN-β production were analyzed and compared to the non-persistent strain MNV-1.CW3. While MNV-S99 and MNV-1.CW3 showed comparable growth characteristics *in vitro*, Stat1 phosphorylation and IFN-β release is strongly decreased after infection with MNV-S99 compared to MNV-1.CW3. In conclusion, our results show that unlike MNV-1.CW3, MNV-S99 establishes a persistent infection in mice, possibly due to interfering with the innate immune response.

## Introduction

The non-enveloped noroviruses constitute a genus within the *Caliciviridae* family, together with the genera of lagovirus, nebovirus, sapovirus, and vesivirus. Noroviruses have a positive-sense single-stranded RNA genome and are divided into seven genogroups [1]. HuNoVs, which are found in genogroups GI, GII, and GIV are causative for 18% of all acute gastroenteritis cases in humans worldwide [2]. The disease is characterized by a rapid onset of typical symptoms, such as nausea, vomiting, watery diarrhea and abdominal pain. In immunocompetent individuals the infection is usually self-limiting and symptoms cease at 48 h to 72 h post infection (hpi). However, immunocompromised individuals can shed these viruses for months or even years after clearance of symptoms [3, 4].

To date, the analysis of pathogenesis and immunity of HuNoV infections is hampered due to the lack of an effective cell culture system and small animal model. However, description of the *in vitro* propagation of HuNoV [5] and of moderate increases of HuNoV titers in a small animal model [6] represent promising first steps toward the development of effective cell culture systems. An alternative to study HuNoV in mice is the investigation of the related MNV. MNV is one of the most prevalent pathogens in colonies of laboratory mice worldwide [7–9]. Unlike HuNoVs, MNVs replicate efficiently in murine macrophages and dendritic cells [10, 11].

The MNV strain MNV-S99 used in this study was isolated in Germany in 2007 [8] from a mouse housing unit, where a prevalence of about 64% of MNV infection in various breeding colonies of immunocompromised, transgenic and wild type mouse lines has been determined.

Of the MNV strains described so far, some have been shown to cause acute infection that is resolved within a few days (e.g. MNV-1 and WU11). The majority of strains however, have been reported to persist in both immunocompetent and immunocompromised mice (e.g. MNV-2, MNV-3, MNV-4, CR1, CR3, CR6, CR7, MNV-O7) [12–17]. Interferon (IFN) production is known as a very rapid and effective mechanism by the host cells to ward off invading pathogens. Secretion of IFN by host cells leads to up-regulation of IFN-stimulated genes (ISGs) in the infected and in neighboring cells. Production of IFN is initiated upon binding of dsRNA by RIG-I-like receptors, which activates NF-κB andIRF3/IRF7 through interaction with MAVS (mitochondrial antiviral signaling protein). These signaling events trigger the induction of type I IFN and other antiviral proteins (e.g. ISG15, Mx-1, CIITA). The binding of released type I IFN to its receptor on uninfected or infected cells recruits Stat1 (signal transducer and activator of transcription 1) and Stat2. After activation of Stat1 and Stat2 by phosphorylation through the Janus kinases JAK1 and Tyk2, phosphorylated Stat1 and Stat2 bind IRF3 (Interferon Regulatory Factor 3) and assemble the ISGF3 (Interferon-stimulated gene factor 3) transcription factor. ISGF3 translocates to the nucleus where it binds the IFN-stimulated response element (ISRE) and promotes transcriptional activation (reviewed in [18]).

Invading pathogens, e.g. vaccinia virus [19], simian virus 5 [20], hepatitis C virus [21–23], rotaviruses [24] and influenza viruses [25] have developed a plethora of mechanisms to evade the host innate immune system. Several studies have shown, that the antiviral proteins Stat1 and IFNs type I and type II, respectively, are essential for the inhibition of MNV replication *in vivo* as well as *in vitro* [10, 14, 26–28]. After infection MNV RNA is recognized by the cellular sensor MDA5 (melanoma differentiation antigen 5). Therefore, mice lacking MDA5 show increased viral titer in the proximal intestine as well as in dendritic cells [29]. Furthermore, IRF3 and IRF7, which are activated during RNA recognition by MDA5 are important for the antiviral response against MNV [30].

Little is known about the mechanisms enabling different MNV strains to evade the host innate immunity and establish persistent infection. A single amino acid exchange was reported in the viral NS1/2 (D94E) non-structural protein of MNV strain CR6 that determines establishment of persistence in immunocompetent mice. The same conservative exchange (D94E) in the non-persistent strain MNV-1.CW3 was sufficient to establish a persistent infection and to increase viral replication in the proximal colon of infected mice. Thus, a contribution of NS1/2 to the viral tropism necessary for persistence was assumed [15]. Furthermore a recently published study has shown that the induction of interferon lambda (IFN-λ) is essential to eliminate a persistent MNV infection [31]. Treatment of WT mice with IFN-λ cures persistent infection with MNV-CR6.

Here, we characterized the persistent MNV strain MNV-S99 in immunocompetent mice in comparison to the non-persistent strain MNV-1.CW3 and its impact on the modulation of Stat1 activation and IFN-β release in the murine macrophage cell line RAW264.7.

## Material and Methods

### Ethics Statement and *in vivo* infection

Animal procedures were performed in accordance with national and European guidelines of the Federation of Laboratory Animal Science Associations (FELASA) and were approved by local authorities (permit # G 0299/07) and by the MPI IB IACUC (Institutional Animal Care and Use Committee of Max Planck Institute for Infection Biology) as well. Employing a specific score sheet to minimize animal distress and suffering animals have been monitored during the 5 weeks experimental procedure 3 times per day. None of the mice have shown any clinical symptoms. For *in vivo* replication, ten 8 week old female wild type mice (C57BL/6J) were orally inoculated with 5 x10^5^ TCID_50_/mice of the strain MNV-S99 and as control 10 mice of the same type, age and gender were inoculated with clarified supernatant from uninfected RAW264.7 cells. Survival of the mice and MNV genome equivalents per mg stool (GE/mg stool) in the feces were monitored for a 35 days period. For quantification of tissue viral loads, mice were sacrificed 35 days post inoculation by cervical dislocation and organs were analyzed for viral genome titers by reverse transcription quantitative real-time PCR (RT-qPCR).

### Cells, viruses and *in vitro* infections

The murine macrophage cell line RAW264.7 (ATCC-TIB-71) was directly obtained from ATCC (Manassas, USA). RAW267.4 cells permissive for MNV infections [11] were cultured in DMEM-HIGH, (DMEM, 10% low endotoxin fetal calf serum (FCS) (GE Healthcare Bio-Sciences, Piscataway, USA) and 2 mM glutamine (Biochrome, Berlin, Germany). Cells were grown at 37°C and 5% CO_2_. Cell passage numbers ranged between 5 and 18 throughout the experiments. Recombinant murine IFN-β was obtained from Sigma-Aldrich (St.Louis, USA). For infection studies, the MNV strains MNV-S99 [8] at passage 3 and the non-persistent strain MNV-1.CW3 at passage 4 (gift from H. Virgin IV, Washington University) were used.

For MNV propagation, RAW264.7 cells were grown in DMEM-HIGH in 175 cm^2^ flasks for 3 days to about 80% confluence. Then the cells were scraped off, centrifuged, re-suspended in 500 μl DMEM-LOW (DMEM, 3% low endotoxin FCS and 2 mM glutamine) and incubated at 37°C with 200 μl of virus suspension for 90 min. Subsequently, 50 ml DMEM-LOW was added and cells were seeded into a 175cm^2^ flask. After appearance of complete cytopathic effect (CPE), cells were frozen and thawed twice and the supernatant was clarified by centrifugation (4°C, 30 min, 3,500xg). Virus stocks were purified through a 30% (w/w) sucrose cushion at 100,000xg for 4 h at 4°C. Virus pellets were re-suspended at 4°C for 16 h in 1 ml DMEM-LOW. One day prior to infection, 1x10^6^ RAW264.7 cells were seeded in 6 cm dishes in DMEM-LOW, and subsequently infected in DMEM-LOW at indicated MOIs (multiplicity of infection) and time points. The viral titer was measured by TCID_50_ titration using the Spearman and Kärber algorithm.

### RNA extraction from feces and tissue from infected mice and quantification

For viral RNA extraction from feces two stool pellets were dispersed in 500 μl PBS overnight. Samples from tissues were homogenized in 500 μl PBS using innuSPEED Lysis tubes P and SpeedMill (Analytic Jena, Jena, Germany). The suspensions were clarified by centrifugation and 140 μl of the supernatant was used for RNA extraction by Qiagen viral extraction kit (Qiagen, Hilden, Germany), according to the manufacturers protocol. Quantification of MNV RNA by RT-qPCR was performed as previously described [8].

### SDS-PAGE and Western blot analysis

Infected RAW264.7 cells were lysed with RIPA buffer (Sigma-Aldrich, St.Louis, USA) containing protease inhibitors cocktail (Roche, Basel, Swiss). The lysates were clarified by centrifugation, subjected to SDS-PAGE, and transferred to PVDF membranes. Membranes were blocked in blocking buffer (Roti®Block, Roth, Karlsruhe, Germany) for 60 min and incubated with a 1:1,000 or 1:200 dilutions of primary antibodies according to the manufacturer’s protocol. The primary antibodies for Stat1 and pStat1 (1:1,000 dilutions) were obtained from NEB (Frankfurt a.M., Germany). The primary antibodies for β-actin (1:200 dilutions) were obtained from Santa Cruz (Dallas, USA), and the secondary antibodies anti-rabbit-HRP and anti-goat-HRP were purchased from Sigma-Aldrich (St. Louis, USA) and used in a 1:10,000 dilution.

### IFN-β and cell based ELISA

The IFN-β levels in supernatants from infected RAW264.7 cells were analyzed using the mouse IFN-β ELISA Kit (PBL, Piscataway, USA) according to the manufacturer’s instructions. Three individual experiments in triplicates were done. The obtained nine data points were used for statistical calculation. The activation of Stat1 was analyzes using the human/mouse-Phospho-Stat1 cell based ELISA (RayBiotech, Norcross, USA) according to the manufacturer’s instructions. Four independent experiments were performed in duplicates.

### Statistics

Statistical analysis was performed using GraphPad Prism version 5.04 for Windows, (GraphPad Software, San Diego, USA). Gaussians distribution of data points were analyzed by using Shapiro-Wilk test. Significances were determined by using either Mann-Whitney-Test or Wilcoxon signed-rank test, p-value ≤ 0.05 considered to be statistically significant.

## Results

### The MNV strain MNV-S99 persists in wild type mice for 5 weeks

**Fig 1:**
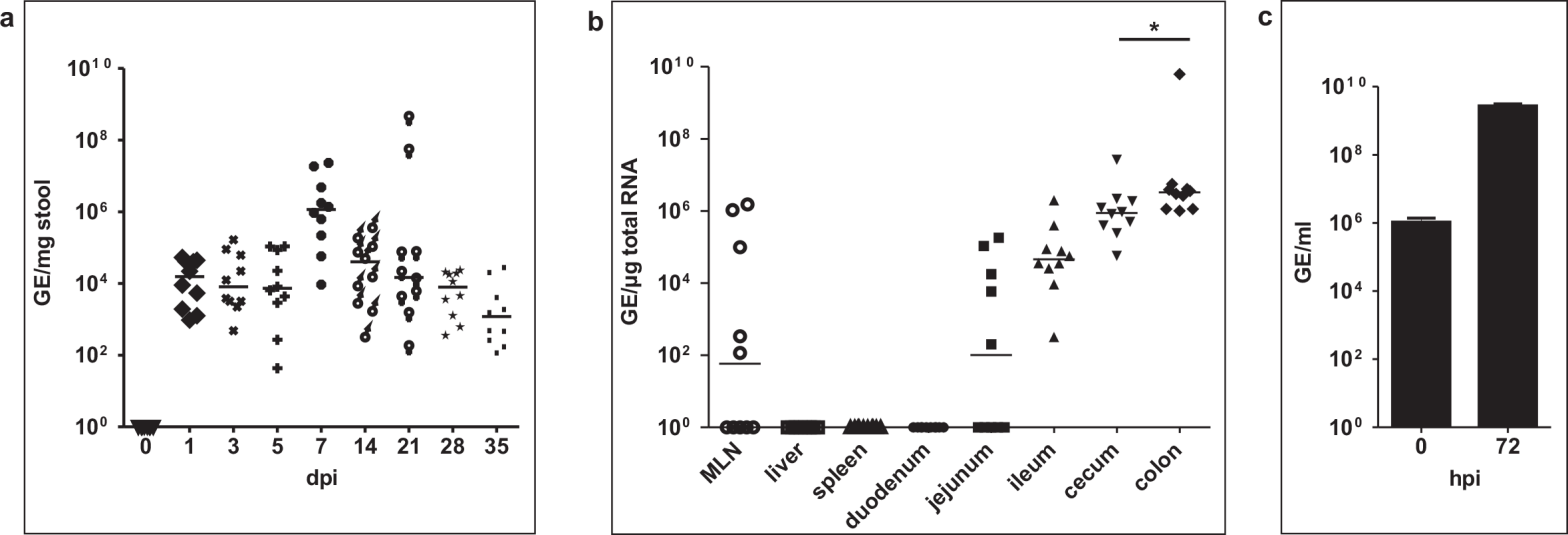
Replication of the persistent strain MNV-S99 occurs mainly in the large intestine. Ten 8 weeks old mice were infected with 5x10^5^ TCID_50_ MNV-S99 per mice. **(a)** Stool samples were collected 0, 1, 3, 5, 7, 14, 21, 28 and 35 dpi. **(b)** Tissues were harvested from MNV-S99 infected mice 35 dpi. Numbers of genome equivalents were measured by quantitative RT-PCR. Horizontal bars in (a) and (b) represent median values. **(c)** RAW264.7 cells were infected with suspension of colon tissue of infected mice. 72 hpi CPE was visible and viral GE/ml in the supernatant were analyzed by quantitative RT-PCR. Statistically significant differences (p ≤ 0.05) were determined by Mann-Whitney-Test and are denoted by asterisks (ns: not significant).

In order to investigate *in vivo* persistence of MNV-S99, immunocompetent mice were infected either with MNV-S99 or with clarified supernatant from uninfected RAW264.7 cells as mock control. All animals were negative for MNV infection at initiation of the experiments (Fig 1A). Mock animals remained RNA negative during the entire course of the experiment (data not shown). Viral shedding was monitored by determining genome titers in the stool every 2 days for the first week and then weekly for another 4 weeks post infection (pi). MNV-S99 infected mice did not shown any sign of gastroenteritis or any other clinical symptoms. One mock infected, MNV-RNA negative mouse died 4 days post infection (dpi) of unknown cause, whereas all MNV-S99 infected mice survived for 5 weeks. At day 1 pi MNV-S99 RNA was detectable in feces of all infected mice. At this time point, viral RNA titers were from 9.5x10^2^ to 5.3x10^4^ GE/mg stool. The highest titer of 2.3x10^7^ GE/mg stool was measured 7 days post infection. During the following 4 weeks viral loads decreased, but all 10 mice were still MNV positive at day 35 pi, displaying viral RNA titers between 1.3x10^2^ to 3.3x10^4^ GE/mg stool (Fig 1A).

These data show that MNV-S99 can persistently infect mice and is shed in feces over a course of 35 days.

### MNV-S99 persists in large and small intestine with exception of duodenum

It was shown that persistence of MNV is linked with a D94E mutation of the NS1/2 non-structural protein and that the replication of persistent MNV strains occurs in the large intestine [12, 15]. The MNV-S99 strain used here also carries this mutation [8]. In order to verify whether MNV-S99 exhibits a tropism comparable to other persistent strains, the viral RNA titers in different organs were analyzed. Following euthanasia of animals 35 dpi, liver, spleen mesenteric lymph nodes (MLN), small intestine (duodenum, jejunum and ileum), cecum and colon were collected and the viral RNA titers in the tissue were determined. MNV RNA was undetectable in all organs of mock-infected control mice. No MNV-S99 RNA was detected in liver and spleen in any of the infected animals (Fig 1B). In 5 out of 10 mice viral RNA could be detected in MLN with titers between 1.3x10^2^ and 1.5x10^6^ GE/μg total RNA. MNV-S99 RNA was undetectable in the duodenum in all infected animals. In contrast 5 out of 10 mice were positive in the jejunum for MNV-S99 RNA, reaching titers from 7.5x10^3^ to 6.7x10^6^ GE/μg total RNA. MNV-S99 RNA was further detected in the ileum, cecum and colon of all infected mice. Furthermore, highest RNA titers were detected in the colon, with mean values of 6.3x10^8^ GE/μg total RNA, followed by cecum (3.5x10^6^ GE/μg total RNA) and ileum (2.7x10^5^ GE/μg total RNA) (Fig 1B).

To test whether the virus isolated from the organs was still infectious, we used a MNV cell culture system. RAW264.7 macrophages were infected with virus suspension obtained from colon tissue of MNV-S99 infected mice. All tested (n = 6) tissue suspensions induced a CPE in RAW264.7 cells (Fig 1C). A significant increase of the viral titers from 1.1x10^6^ to 2.7x10^9^ GE/ml was determined in the supernatant at 72 hpi (p = 0.0022) showing that there is viable and replication-competent virus in the colon. The data show that the tropism of MNV-S99 is similar to other persistent MNV strains. The replication occurs mainly in the large intestine, but MNV-S99 RNA was also detectable in parts of the small intestine, jejunum and ileum.

### Growth comparison of MNV-S99 and MNV-1.CW3 in RAW264.7 cells

In order to characterize MNV-S99 in more detail and to gain more insights into immune signaling, its replication kinetics was compared to the non-persistent control strain MNV-1.CW3 in RAW264.7 macrophages.

**Fig 2:**
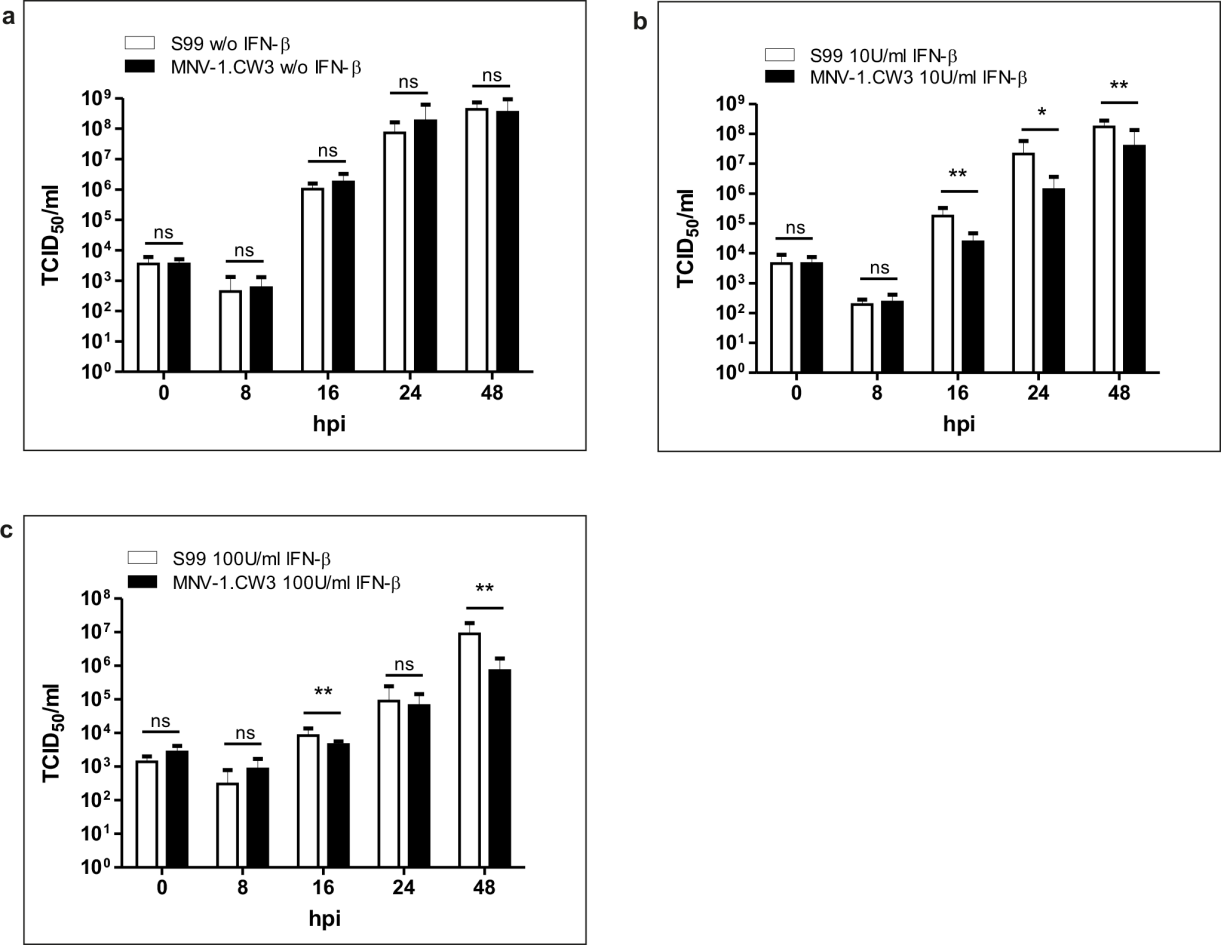
The persistent strain MNV-S99 is more resistant to antiviral effect of IFN-β, compared to MNV-1.CW3. RAW264.7 cells were infected with MNV-S99 or MNV-1.CW3 using an MOI of 0.1 TCID_50_/cell **(a)** without or in the presence of **(b)** 10 U/ml IFN-β or **(c)** 100 U/ml IFN-β. Viral titers in cell culture supernatant were measured by virus titration using the Spearman and Kärber algorithm to calculate the TCID_50_/ml. Three independent experiments, performed in triplicates were done. Statistical analysis was done by using Wilcoxon signed-rank test, statistically significant differences (p ≤ 0.05) are denoted by asterisks. Dashed line represents the detection limit of the assay.

Cells were infected with a MOI of 0.1 TCID_50_/cell and viral titers were determined in the cell culture supernatant at indicated time points. Eight hours post infection the measured viral particles in the supernatant of both strains were decreased probably corresponding to the virus eclipse phase. After 16 hpi the mean of viral titer of MNV-S99 and MNV-1.CW3 increased to 1.0x10^6^ TCID_50_/ml and 1.8x10^6^ TCID_50_/ml, respectively. Complete CPE was visible at 48 hpi, exhibiting maximum mean titers of 4.8x10^8^ TCID_50_/ml and 3.5x10^8^ TCID_50_/ml, respectively. Both strains replicated with the same efficiency *in vitro* (Fig 2A).

### MNV-S99 is less sensitive to Interferon-β than MNV-1

Previous studies have shown that the replication of the non-persistent strains MNV-1.CW1 and MNV-1.CW3 are inhibited by type I and type II IFNs [26, 27, 30].

To verify the anti-viral activity of type I IFN to the replication efficiency of MNV-S99, we infected RAW264.7 cells with an MOI of 0.1 TCID_50_/cell without and in the presence of 10 and 100 U/ml IFN-β, and compared the replication rate of MNV-S99 to the control strain MNV-1.CW3. IFN-β inhibited the replication of MNV-S99 and MNV-1.CW3 in a time and dose dependent manner (Fig 2B and 2C). Analysis of the replication efficiencies of both strains in the presence of 100 U/ml IFN-β showed that MNV-1.CW3 infection is more sensitive to IFN-β compared to MNV-S99 (Fig 2C). At 48 hpi the mean viral titer of MNV-S99 was 1.8x10^7^ TCID_50_/ml, whereas the mean titer of MNV-1.CW3 was significantly lower (7.2x10^5^ TCID_50_/ml, p = 0.0039). Treatment with 10 U/ml IFN-β determined a moderate decrease of viral titers compared to infection with 100 U/ml IFN-β (Fig 2B and 2C). Stimulation with the lower amount of IFN-β revealed also a significant difference (p = 0.0039) of mean viral titers at 48 hpi between MNV-S99 and MNV-1.CW3 with 1.7x10^8^TCID_50_/ml and 3.9x10^7^ TCID_50_/ml, respectively (Fig 2B). Similar effects were observed using viral RNA quantification instead of TCID_50_ determination (data not shown). These data show that despite a nearly identical replication efficiency of both strains the persistent strain MNV-S99 is significantly more resistant against the antiviral effect of IFN-β than the non-persistent MNV-1.CW3.

### Interferon-β release is strongly decreased after MNV-S99 infection

**Fig 3:**
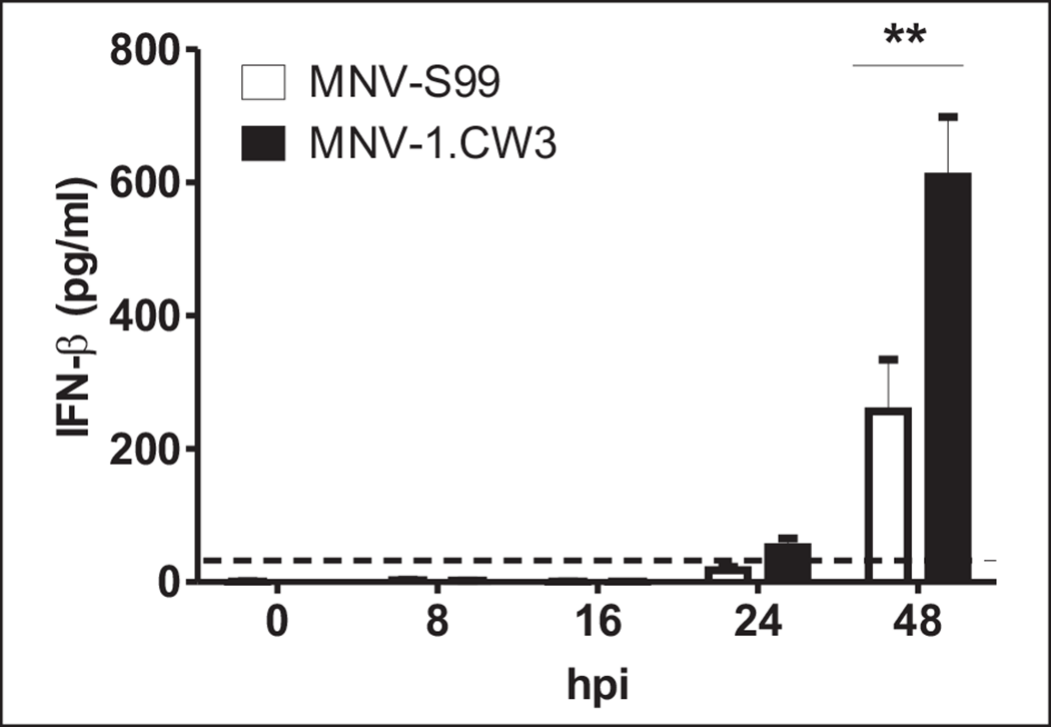
Interferon-β release is strongly decreased after MNV-S99 infection. RAW264.7 cells were infected with MNV-S99 or MNV-1.CW3 strains at a MOI of 0.1 TCID_50_/per cell for indicated time points. The concentration of IFN-β in cell culture supernatants was determined by mouse IFN-β ELISA. Three independent experiments, performed in triplicates were done. Statistical analysis was done by using Wilcoxon signed-rank test, statistically significant difference (p ≤ 0.05) is denoted by asterisk. The dashed line represents the detection limit of the assay.

Delay of IFN-β production and / or release is a potent mechanism used by many viruses to avoid host immune defense mechanisms [21, 22, 32, 33]. To examine whether MNV-S99 infection indeed decreases IFN-β release, RAW264.7 macrophages were infected either with MNV-S99 or with MNV-1.CW3 (MOI 0.1 TCID_50_/cell). Murine IFN-β release to the supernatant was analyzed by ELISA at indicated time points (Fig 3). IFN-β release was first detected at 24 hpi for both MNV-S99 and MNV-1.CW3 infections, while the highest concentration of IFN-β was measured at 48 hpi. However, overall extracellular IFN-β levels were significantly (p = 0.0039) lower during MNV-S99 infection compared to MNV-1.CW3 (271.6 pg/ml and 608.9 pg/ml, respectively). This suggests that infected cells fail to efficiently induce IFN-β production and / or secretion in the presence of MNV-S99 which might contribute to establishing persistent infections with this strain.

### MNV-S99 fails to induce Stat1 phosphorylation in RAW264.7 cells

**Fig 4:**
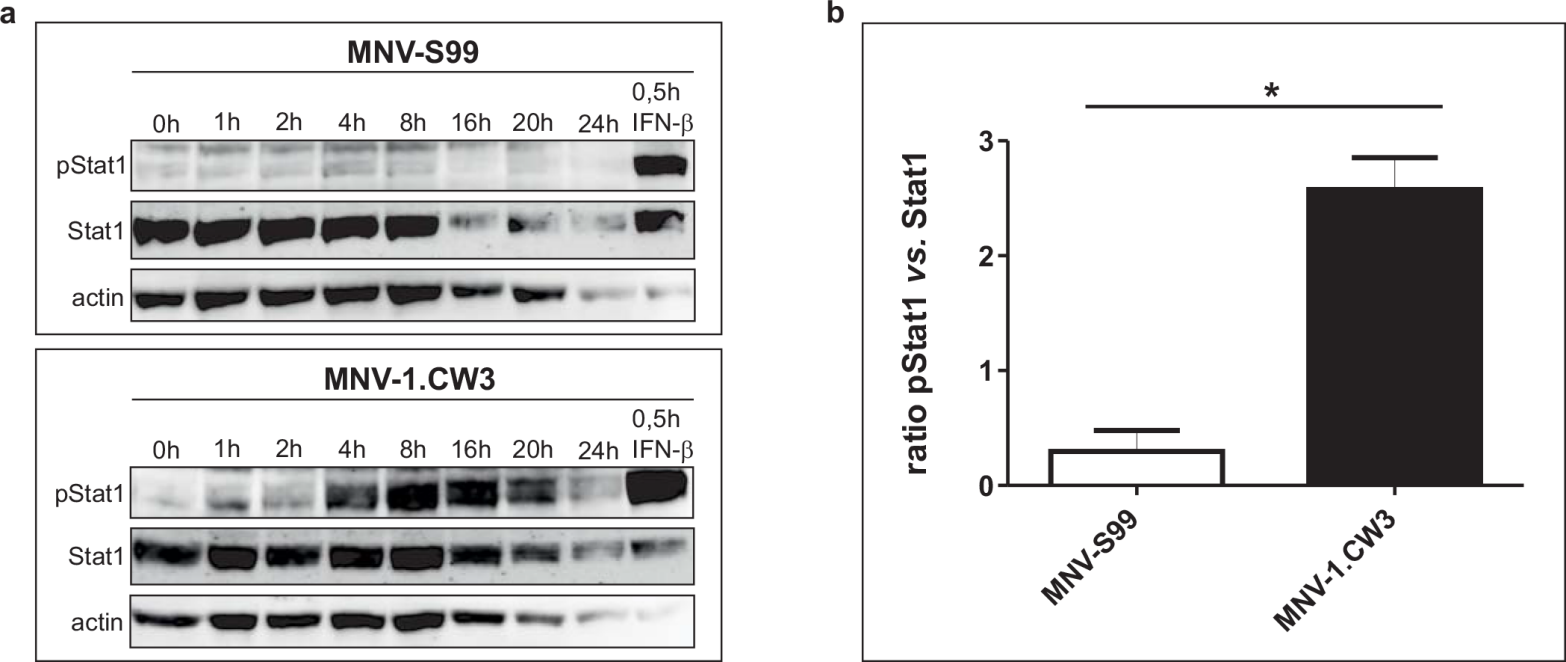
Stat1 activation is strongly decreased by MNV-S99 infection. **(a)** RAW264.7 cells were infected either with MNV-S99 or MNV-1.CW3 strains using a MOI of 1 TCID_50_/cell for indicated time points. Cells were lysed with RIPA buffer, phosphorylation of Stat1 was analyzed by western blot analysis. As positive control for Stat1 phosphorylation, RAW264.7 cells were treated with 100 U/ml IFN-β for 30 minutes. **(b)** RAW264.7 cells were infected with MNV-S99 or MNV-1.CW3 using a MOI of 0.1 TCID_50_/cell for 16 h. Activation of Stat1 was measured by cell based ELISA assay. Activation was measured by the ratio of phosphorylated *vs*. non-phosphorylated Stat1. Data points were calculated as fold change of Stat1 activation after 16 hpi relative to mock infected cells. Four independent experiments were performed in duplicate. Statistically significant difference (p ≤ 0.05) was determined by Mann-Whitney-Test and is denoted by the asterisk.

Another explanation for the apparent partial resistance of MNV-S99 to IFN-β is a decrease in the IFN-signaling events. After binding to IFN-receptors, the signal is transduced to the interior of the cell via the JAK/Stat pathway. It has previously been reported that MNV-1 causes lethal infection in Stat1^-/-^ mice [10]. To determine whether Stat1 is differentially activated after MNV-S99 or MNV-1.CW3 infection, phosphorylation of Stat1 at different time points was analyzed by western blot analysis. Upon infection of cells with MOI of 0.1 TCID_50_/cell, phosphorylation of Stat1 was undetectable by Western blot analysis after infection with both strains. Therefore, RAW264.7 cells were infected with MOI of 1 TCID_50_/cell. As positive control for Stat1 activation, RAW264.7 cells were stimulated for 30 minutes with 100 U/ml IFN-β. We consistently detected Stat1 phosphorylation by MNV-1.CW3 infection from 4 hpi to 20 hpi (Fig 4A lower panel). In contrast to the robust Stat1 activation by MNV-1.CW3, Stat1 activation was undetectable in cells infected with MNV-S99 at all analyzed time points (Fig 4A, upper panel). For both strains, the cells started to round up and detach 16 hpi indicative of CPE at the MOI of 1.

To confirm these results, Stat1 activation was additionally analyzed by cell based ELISA. RAW264.7 cells were infected either with MNV-S99 or with MNV-1.CW3 using an MOI of 0.1 TCID_50_/cell for 16 hours. The activation was calculated by the ratio of phosphorylated to non-phosphorylated Stat1 signals (Fig 4B). The non-persistent strain MNV-1.CW3 led to activation of Stat1 whereas the Stat1 phosphorylation was significantly decreased after infection with MNV-S99 (p = 0.0148). This observation confirmed our Western blot analysis results indicating that Stat1 is not activated by infection with the persistent strain MNV-S99 in contrast to MNV-1.CW3 infection.

## Discussion

HuNoVs can establish persistent infections in immunocompromised patients, which can shed virus for over 2 years [3, 4, 34]. However, the mechanisms leading to persistence remain largely unknown, mainly due to the lack of suitable model systems. MNVs are a valuable model virus to study the pathomechanisms of norovirus infections. MNV can replicate *in vitro* and can be used to infect mice [10, 11]. Since the discovery of the MNV in 2003, many different MNV strains have been described exhibiting different molecular and virulence properties. Until now, little is known about the mechanisms that enable some MNV strains to establish persistent infections. A previous study showed that persistence of MNV is linked with a mutation in the NS1/2 non-structural protein [15]. All MNV strains which were able to establish a persistent infection showed an amino acid exchange at position 94 (D94E) of this protein. The introduction of this mutation into the MNV-1 strain, which is usually cleared by the immune system within days, established a persistent infection in immunocompetent mice [15]. In the present study, we have used this MNV model to characterize the MNV strain MNV-S99 in more detail. *In vivo* experiences revealed that MNV-S99 establishes persistent but asymptomatic infection in WT mice for at least 5 weeks. Different studies reported that replication of MNV after a persistent infection mainly occurs in the colon and cecum [12, 15]. The data shown here support the observation that replication of persistent MNV strains is established in the large intestine.

The used MNV strain, MNV-S99 revealed the presence of the D94E mutation [8], corroborating the observation by Nice et al. that this mutation is linked with the property of MNV strains to establish a persistent infection [15]. This mutation is found in almost all MNV strains, with the exception of MNV-1 related strains, whose infections are cleared within days by the host innate immune system. Our *in vivo* experiments have shown that MNV-S99 replicates mainly in the large intestine of immunocompetent mice. These data supported the observation that the D94E mutation is also required for the colonic tropisms of persistent MNV replication in mice beside its role in establishing persistent infections [15].

The MNV strain WU11 causing acute infection also shows this persistence-associated mutation. In contrast to persistent strains WU11 can be eliminated by the host immune system within days [17]. However, so far it is unknown why infection by WU11 can be cleared within days.

As previously described, type I and type II IFNs can efficiently block the replication of MNV [26–28, 30]. However, the underlying mechanisms are not well understood. In this study, no significant differences in the viral replication in RAW264.7 cells between the persistent MNV-S99 and non-persistent MNV-1.CW3 were observed. The results showed that the response to type I IFN was significantly different. Comparison of MNV-S99 and MNV-1.CW3 replication under treatment with 10 U/ml IFN or with 100U/ml IFN-β revealed that the propagation of the non-persistent strain MNV-1.CW3 was inhibited significantly more and viral titers were lower 48 hpi as compared to MNV-S99 infections (Fig 2). These results are in line with the published observation that the replication of MNV is inhibited by IFNs. However, the replication of MNV-S99 seems to be more resistant against the antiviral effect of IFN-β compared to MNV-1.CW3. This mechanism may enable MNV-S99 to evade the host immune system thus establishing persistent infections.

Previous reports have shown that the infection with different non-persistent MNV strains cause the release of type I and type II IFNs *in vivo* and *in vitro* [14, 29, 30, 35–37]. After viral infection, IFN is secreted from cells. The released IFN can either bind to the receptor of uninfected neighboring cells, thus inducing the antiviral state of these cells, or to the receptor of infected cells, enforcing the IFN release via the IRF7 dependent positive feedback loop [38].

The release of IFN-β after infection with MNV-S99 and MNV-1.CW3 was detectable in the cell culture supernatants starting 24 hpi. Highest IFN-β concentrations were measured at 48 hpi when the complete CPE was visible. These data showed that the infection with MNV-1.CW3 resulted in a robust induction of IFN-β production. In contrast, the infection with the persistent strain MNV-S99 induced a significantly lower amount of IFN-β (Fig 3).

In 2011, a novel open reading frame 4 (ORF4) was identified [36] which was found in all known MNV strains and isolates, but not in any HuNoV. ORF4 encodes for a non-structural protein, called virulence factor 1 (VF1). It was demonstrated that VF1 affects the innate immune system by suppression of the release of IFN-β after infection with MNV [36]. Our data showed a reduced IFN-β release after infection with MNV-S99, compared to MNV-1.CW3. It might be possible, that the expression level of the VF1 protein is different between MNV-S99 and MNV-1.CW3. A higher VF1 expression in the case of a MNV-S99 infection would cause a decrease IFN-β release. Unfortunately, we were unable to detect any VF1 protein expression in MNV-S99 or MNV-1.CW3 infections, which may be due to its high isoelectric point (personal communication, Ian Goodfellow). Further investigations are necessary to investigate the specific function of the VF1 protein in the decreased IFN-β release in MNV-S99 infection.

In 2015 it has been shown that the inhibition of IFN-λ induction is also required for MNV persistence [31]. This study revealed that the persistent MNV strain CR6 fails to induce IFN-β as well as IFN-λ in WT mice. Treatment of the mice with IFN-λ curves the persistent infection within few days. Despite the failure of induction of IFN-β *in vivo*, *in vitro* investigation revealed an induction of IFN-β after infection with MNV CR6 comparable with that of the non-persistent control strain [31]. In contrast, our data show that the induction of IFN-β *in vitro* is significant lower, compared to the non-persistent strain MNV-1.CW3. Further studies are needed to determine the *in vivo* role of IFN-λ and IFN-β in the establishment of a persistent infection by MNV-S99.

Suppression of IFN signaling is a common mechanism for many gastroenteritis viruses to evade the host immune system [39, 40]. Infections with the norovirus related feline calicivirus (FCV) induce the release of type I IFN in a strain dependent manner. FCV-2280 strain resulted in a robust release of IFN-β, whereas other FCV strains failed the induction of IFN-β production after infection [41]. A recently published study has shown that the FCV non-structure protein p39 (family of putative viral helicases) suppresses the IFN-β production by preventing IRF3 activation after infection [42].

Beside IFNs other host proteins have been identified as important factors for the elimination of MNV infections. The essential role of Stat1 for the efficient clearance of MNV infections by the host immune system was shown earlier. Stat1 limits the viral replication in the intestine and prevents apoptosis in intestinal cells [10, 14]. IRF3 and IRF7 are known to inhibit the viral replication [14]. MNV RNA is detected and bound by MDA5 [29]. Activated MDA5 binds to the adaptor molecule MAVS, resulting in a production of type I IFN. Infections of primary dendritic cells of MDA5^-/-^ mice led to an increase of viral titer of MNV and a decrease of IFN release [29]. Consistent with these previous findings we showed here that infection with the non-persistent strain MNV-1.CW3 leads to phosphorylation of Stat1. In contrast, after infection with the persistent strain MNV-S99, no phosphorylation of Stat1 was detectable.

In summary, using our mouse model system we could show that the MNV strain MNV-S99 establishes persistent infection in immunocompetent mice. This persistent strain replicates efficiently in the large intestine. *In vitro* investigations have shown that MNV-S99 is more resistant to the antiviral effect of IFN-β compared to the non-persistent strain MNV-1.CW3. This may be an advantage for the virus to evade the host innate immune system. Here we have shown for the first time that MNV-S99 failed to induce Stat1 activation and subsequently caused a much lower IFN-β release. The suppression of Stat1 activation may enable MNV-S99 to overcome the host innate immune system and establish persistent infections in mice.

MNV-S99 and MNV-1.CW3 show amino acid sequence diversity between 95.8% in ORF1 and 90.1% in ORF3. Consistent with this divergence, our investigations have shown that both viruses can cause different phenotypes *in vitro* and *in vivo*. However, further studies are needed to identify the mechanisms leading to the observed differences in Stat1 activation and IFN-β sensitivity. MNV-S99 is able to establish persistent infections in wild type mice, whereas other strains will be eliminated within days, like MNV-1. A limitation of our study is that the *in vivo* experiments were done only once. Despite this limitation our data show for the first time that MNV-S99 can establish a persistent infection mainly in the large intestine in WT mice. The *in vivo* replication of MNV-S99 was expectable, however; up to now all described MNV strains established a persistent infection, with the exception of MNV-1 related strains and MNV-WU11 [12–17, 31]. In addition MNV-S99 carries the D94E mutation which has been described previously to be associated with persistence in WT mice [15]. Due to the importance of reducing animal usage, we renounced the repeating of the *in vivo* experiments. Understanding the functional contribution of the innate immune system in efficient clearance of norovirus infections is important to identify innovative therapy options for the elimination of persistent norovirus infections in humans.
